# Experiments with Bichloride of Mercury

**Published:** 1893-10

**Authors:** Carrie M. Stewart

**Affiliations:** Ann Arbor, Mich.


### EXPERIMENTS WITH BICHLORIDE OF MERCURY.[1]

BY CARRIE M. STEWART, D.D.S., ANN ARBOR, MICH.

Because of the peculiar action of bichloride of mercury upon material of an albuminous nature, its efficacy as a germicide is believed by some scientists to be less than the standard usually assigned to it. When brought in contact with the substance to be acted upon, the albumen present is superficially coagulated, the interior of the mass escaping many times, because of a lack of penetrating power in the disinfectant. Such being the case, it has been urged that in the action of bichloride of mercury upon the germs of disease, the same effect is produced,—*i.e.*, the external gelatinous capsule of the germ is coagulated, while the internal, vital portion is left untouched, and if by any means the compound between this covering and the disinfectant be dissolved, the micro-organism is quite as ready to manifest its characteristic phenomena as though it had never been subjected to the action of the disinfectant. Acting upon this hypothesis, investigators have endeavored by experimental research to conclusively confirm or disprove the theory, but as yet their efforts have not met with success. A method which

---

[1] Read before the World's Columbian Dental Congress, Chicago, August, 1893.

seems very satisfactory so far as showing the effect of certain chemical substances upon the supposed compound formed between the bichloride of mercury and the envelope of the germ, is that used by Dr. McClintock, author of a paper entitled "Corrosive Sublimate as a Germicide." This method has been used in the experiments given here, and briefly outlined is as follows:

An ordinary test-tube is plugged with cotton-wool, and through this plug is passed a glass rod containing a plug of the same material for part of its length. In the test-tube is a small amount of non-germicidal alkali, as calcium carbonate or carbonate of sodium and sodium chloride. After sterilization the apparatus is ready for use. To a definite quantity of the bouillon culture of the germ was added a definite amount of the $HgCl_2$, strength 1 to 1000. After a given length of time one c.c. of the disinfected culture was transferred to the prepared test-tube and connected with a hydrogen sulphide generator until sufficient precipitation of the mercury had taken place. Gelatin or agar was then inoculated with from three to four loops of the preparation, and Petri plates made. The chemical action taking place is very manifest, the hydrochloric acid formed being neutralized by the alkali present, sodium carbonate or calcium carbonate. The sodium chloride solution assists in dissolving the compound formed between the bichloride of mercury and the gelatinous material, or the protecting envelope of the micro-organism.

The germs used in experimenting were the staphylococcus pyogenes aureus and albus, because of their frequent occurrence, and, although not spore-forming in the common acceptance of the term, they are nevertheless very resistant to destructive influences. This work was done to satisfy myself of the efficacy of bichloride of mercury as a germicide under certain conditions, and although the experiments made were comparatively few in number they were accomplished with all possible care and accuracy.

April 7, 1893, five c.c. of 1 to 1000 bichloride of mercury to five c.c. of bouillon culture of staphylococcus pyogenes aureus, three days old. Time of exposure, fifteen, thirty, sixty minutes. Agar plates. April 11, the fifteen- and thirty-minute plates well developed. April 27, sixty-minute plate still undeveloped. April 28, one c.c. of $HgCl_2$ to five c.c. of culture of the staphylococcus pyogenes albus, fifteen days old. Time, twenty-four hours. Three agar plates made. May 8, all three plates developed. April 28, five c.c. of $HgCl_2$ to five c.c. of culture of albus, fifteen days old. Time, twenty-four hours. Three agar plates made. May 8, no development on any plate. May 3, fifteen c.c. of $HgCl_2$ to five c.c. of culture of the albus, five days old. Time, twenty-four hours. Three gelatin plates made. May 11, one colony on each of two plates, the other one being undeveloped. May 3, ten c.c. of $HgCl_2$ to five c.c. of a bouillon culture of the aureus, four days old. Time, twenty-four hours Three gelatin plates made. May 11, no development. May 9, twenty c.c. of $HgCl_2$ to five c.c. of culture of the albus, seven days old. Time, seventy, eighty-five, one hundred, and one hundred and twenty minutes. Two plates for each exposure. May 18, no colonies on the plates of the first two exposures. One of the plates having the one hundred minutes' exposure exhibited one colony, while the other had none.

Of the plates having the two hours' exposure, one plate was undeveloped, while the other possessed three colonies.

May 11, five c.c. of $HgCl_2$ to five c.c. of the aureus culture, two days old. Time, fifteen, thirty, forty-five, and sixty minutes. Agar plates. May 22, one colony on the plate having the forty-five minutes' exposure, none on the others. May 15, two c.c. of $HgCl_2$ to five c.c. of a culture of the albus, seven days old. Time, fifteen, forty-five, and sixty minutes. Gelatin plates. May 22, plate of the fifty-five minutes' exposure very well developed; others not.

All these experiments were performed with sodium carbonate used as a neutralizing agent, care being taken that a sufficient quantity was used to serve the purpose. In the succeeding experiments barium carbonate was used instead.

May 16, five c.c. of $HgCl_2$ to five c.c. of bouillon culture of aureus, six days old. Time, fifteen, thirty, forty-five, and sixty minutes. Gelatin plates. May 26, no development in any plate. May 17, two c.c. of $HgCl_2$ to five c.c. of a culture of the albus, seven days old. Time, twenty-four hours. Gelatin plate. May 24, one colony. May 23, five c.c. of acid solution of $HgCl_2$, 1 to 1000, to five c.c. of culture of the aureus, two days old. Time, five, ten, fifteen, thirty, and sixty minutes. Gelatin plates. June 6, no development in any plate. May 31, one c.c. of acid solution of $HgCl_2$ to five c.c. of albus, six days old. Time, one, five, ten, fifteen, and sixty minutes. Gelatin plates. June 9, no development. June 1, three c.c. of acid solution $HgCl_2$ to five c.c. of culture of albus, nine days old. Time, one, five, fifteen, and thirty minutes. Gelatin plates. June 9, no development.

A little experimenting was done with a solution of $HgCl_2$, 1 to 500, to test its germicidal powers.

When precipitation of the mercury by hydrogen sulphide occurred, the solution was found to be valueless as a germicide; while without the action of the hydrogen sulphide its effect upon the germs was immediate.

May 19, one c.c. of $HgCl_2$, 1 to 500, to five c.c. of albus culture, four days old. Time, one, five, ten, and fifteen minutes. ($H_2S$ employed.) May 22, all profusely developed excepting the plate of the ten minutes' exposure, which had no colonies. May 19, one c.c. $HgCl_2$ to five c.c. of aureus culture, three days old. Gelatin plates. Time, one, five, ten, and fifteen minutes, without precipitation by $H_2S$. May 31, no development in any plate. May 23, five c.c. of $HgCl_2$ to five c.c. of culture of the albus, four days old. (Precipitation of mercury by $H_2S$.) Time thirty minutes. Gelatin. May 31, very well developed.

In looking over the results presented here, one will notice now and again a seeming irregularity in the development of a plate; for instance, a plate of fifteen minutes' exposure may exhibit no colonies, while one of sixty minutes, of the same series, may be well developed. This may be explained by the supposition that only the more feebly resistant germs were transferred to the gelatin, in the first case, and being destroyed by the germicide before the transferrence, no development could consequently take place.

In the latter case a sufficient number of the more resistant germs were brought over to show development under suitable conditions.

Again, enough of the germicide may have been taken over with the germs to have formed a sterilized area around the micro-organisms, preventing their growth in the one case, while in the other the disinfectant was sufficiently disturbed throughout the gelatin to prevent anything of the kind. Care was taken to accomplish this in every case, however.

The neutralization of the acid formed was also especially looked after.

In comparing the results obtained from the use of the ordinary solution of bichloride of mercury and that of the acid solution, it will be noticed that the latter is far more prompt and efficient in its action than the former, due probably to the excess of hydrochloric acid in the acidulated solution.

Although it is manifestly evident that bichloride of mercury is not sufficiently penetrating in its action to serve as a germicide, excepting under conditions most favorable to its complete performance of the results desired, yet the ideal germicide has not been discovered, and those that are so efficient as bichloride of mercury are hard to find. It would seem from the experiments of others, that the more concentrated solutions of this disinfectant are still less to be relied upon in their action than the strengths more ordinarily used, from the fact that the combination between the bichloride of mercury and the capsule of the germ is more quickly effected, and, although the portion influenced is probably of a firmer consistency than that brought about by using a solution of weaker strength, still the amount of penetration is not so great and the desired results not so nearly accomplished. Because of its extremely poisonous nature, bichloride of mercury will never be extensively used alone in dentistry; yet, with a proper knowledge of its powers and those of other germicidal agents of a high degree of usefulness, a combination may be effected in the future which may prove of the utmost value to the oral surgeon.

